# 4,6-Dimethyl-2-thioxo-1,2-dihydro­pyrimidin-3-ium chloride–thio­urea (1/1)

**DOI:** 10.1107/S1600536809016857

**Published:** 2009-05-14

**Authors:** Papa Aly Gaye, Aliou Hamady Barry, Mohamed Gaye, Aminata Diasse Sarr, Abdou Salam Sall

**Affiliations:** a Département de Chimie, Faculté des Sciences et Techniques, Université Cheikh Anta Diop, Dakar, Senegal; b Département de Chimie, Faculté des Sciences, Université de Nouakchott, Nouakchott, Mauritania

## Abstract

In the title compound, C_6_H_9_N_2_S^+^·Cl^−^·CH_4_N_2_S, the 4,6-di­methyl-2-thioxo-1,2-dihydro­pyrimidin-3-ium cation is proton­ated at one of the pyrimidine N atoms. The cations are bridged by the chloride anions through a pair of N—H⋯Cl hydrogen bonds. The amino groups of each thio­urea adduct inter­act with the chloride anions through a pair of N—H⋯Cl hydrogen bonds and the S atom of another thio­urea adduct through a pair of N—H⋯S hydrogen bonds. These inter­actions result in a layered hydrogen-bonded network propagating parallel to the *bc* plane. Except for two H atoms, all atoms are on special positions.

## Related literature

For related structures, see: Seth & Sur (1995 ▶); Jianqiang *et al.* (2006 ▶). For bond-length data, see: Arslan *et al.* (2004 ▶); Hemamalini *et al.* (2005 ▶).
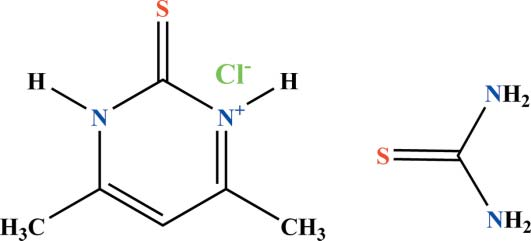


## Experimental

### 

#### Crystal data

C_6_H_9_N_2_S^+^·Cl^−^·CH_4_N_2_S
*M* *_r_* = 252.78
Orthorhombic, 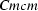

*a* = 6.6459 (4) Å
*b* = 21.6144 (14) Å
*c* = 8.3878 (5) Å
*V* = 1204.88 (12) Å^3^
*Z* = 4
Mo *K*α radiation
μ = 0.63 mm^−1^
*T* = 293 K
0.10 × 0.10 × 0.10 mm

#### Data collection

Nonius KappaCCD diffractometer
Absorption correction: none
1080 measured reflections
636 independent reflections
447 reflections with *I* > 2σ(*I*)
*R*_int_ = 0.024

#### Refinement

*R*[*F* ^2^ > 2σ(*F* ^2^)] = 0.055
*wR*(*F* ^2^) = 0.175
*S* = 1.05
636 reflections
49 parameters
H-atom parameters constrained
Δρ_max_ = 0.44 e Å^−3^
Δρ_min_ = −0.51 e Å^−3^

### 

Data collection: *COLLECT* (Nonius, 1998 ▶); cell refinement: *DENZO*/*SCALEPACK* (Otwinowski & Minor, 1997 ▶); data reduction: *DENZO*/*SCALEPACK*; program(s) used to solve structure: *SHELXS97* (Sheldrick, 2008 ▶); program(s) used to refine structure: *SHELXL97* (Sheldrick, 2008 ▶); molecular graphics: *PLATON*/*PLUTON* (Spek, 2009 ▶); software used to prepare material for publication: *SHELXL97*.

## Supplementary Material

Crystal structure: contains datablocks I, global. DOI: 10.1107/S1600536809016857/er2066sup1.cif

Structure factors: contains datablocks I. DOI: 10.1107/S1600536809016857/er2066Isup2.hkl

Additional supplementary materials:  crystallographic information; 3D view; checkCIF report

## Figures and Tables

**Table 1:** Hydrogen-bond geometry (Å, °)

*D*—H⋯*A*	*D*—H	H⋯*A*	*D*⋯*A*	*D*—H⋯*A*
N1—H1⋯Cl1^i^	0.86	2.46	3.310 (4)	171
N2—H2*A*⋯Cl1	0.86	2.50	3.297 (5)	154
N2—H2*B*⋯S2^ii^	0.86	2.49	3.347 (5)	173

Symmetry codes: (i) 
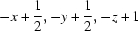
; (ii) 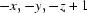.

**Fig. 1.**
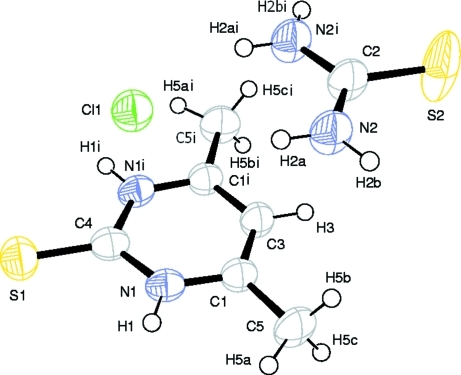
An ORTEP view of the asymmetric unit of the title compound, showing the atom-numbering scheme. Displacement ellipsoids are plotted at the 50% probability level. Symmetry code: (i) -x, y, -z + 1/2

**Fig. 2.**
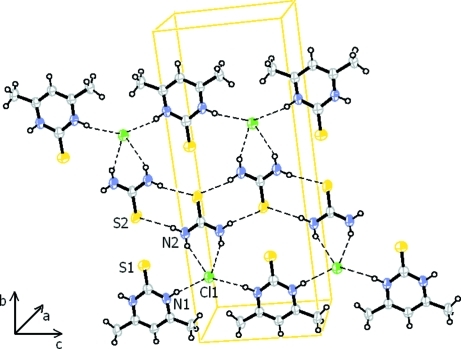
Molecular representation of the compound showing hydrogen bonds. The broken lines stand for hydrogen bonds.

## supplementary crystallographic information

### Comment

The title compound, C_6_H_9_N_2_S.CH_4_N_2_SCl, was characterized by ^1^H and ^13^C NMR, solid-state IR and X-ray crystallographic techniques. The X-ray structure determination reveals that the compound crystallizes in the orthorhombic space group Cmcm with a protonated molecular moiety, a chloride anion and one thiourea adduct in the asymmetric unit.  The molecular geometry is illustrated in Fig. 1. The C—S bond length of 1.649 (7) Å in the molecular adduct and 1.698 (8) Å in the thiourea are double bonds character and are comparable to those observed for 1-(biphenyl-4-carbonyl)-3-p-tolyl-thiourea [1.647 (3) Å for C—S (Arslan et al., 2004)]. The C—N bond lengths are in the range [1.322 (6)-1.371 (6) Å] and are shorter than the double C—N bond length (Hemamalini et al. <i/>, 2005). All atoms, except H5B and H5C, lie on a mirror plane, similar to the observed structure of 4,6-dimethylpyrimidine-2(1H</>)-thione (Seth & Sur, 1995). The molecular adduct forms hydrogen bonds with two chloride anions by  N1—H1···Cl1(-x + 1/2, -y + 1/2, -z + 1) (Fig. 2). Each thiourea molecule is linked to two other thioura molecule by hydrogen bonds and one chloride anion respectively by N2—H2B···S2(-x, -y, -z + 1) and N2—H2A···Cl1 (Table. 2).

### Experimental

Thiourea (2 g, 26 mmol) was reacted with 2,4-pentadione (2.6 g, 26 mmol) in C_3_H_6_O (20 ml) solution, to give the corresponding 1:1 adduct after two hour under refluxing. After cooling to room temperature, 3.4 ml HCl 10*M* was added dropwise to the solution and the resulting mixture was refluxed for one hour before left standing overnight. The filtrate gave yellowish crystal suitable for X-ray analyses after four days of slow evaporation. Yield: 87.69%. m.p. 190±2 °C. Anal. Calc. for C_7_H_13_N_4_S_2_Cl (%): C, 33.26; H, 5.18; N, 22.16. Found: C, 33.37; H, 5.15; N, 22.25. Selected IR data (cm^-1^, KBr pellet): 1599 (ν C═N), 1187 (ν C═S). ^1^H NMR (200 MHz, D_2_O, δ, p.p.m.): 2.40 (s, 6H, –CH_3_); 6.83 (s, 1H, –CH). ^13^C NMR (200 MHz, D_2_O, δ, p.p.m.): 19.26 (–CH_3_); 118.32 (–CH); 168.02 (N═C); 172.90 (N═C—S—H).

### Refinement

The H atoms of the NH_2_ groups were located in the Fourier difference maps and refined by riding motion. Others H atoms were placed geometrically and refined with a riding model. *U*_iso_(H) for H was assigned as 1.2*U*_eq_ of the attached C atoms (1.5 for methyl C atoms).

### Crystal data

**Table:**

C_6_H_9_N_2_S^+^·Cl^−^·CH_4_N_2_S	*F*(000) = 528
*M**_r_* = 252.78	*D*_x_ = 1.393 Mg m^−^^3^
Orthorhombic, *C**m**c**m*	Mo *K*α radiation, λ = 0.71073 Å
Hall symbol: -C 2c 2	Cell parameters from 653 reflections
*a* = 6.6459 (4) Å	θ = 1.0–25.4°
*b* = 21.6144 (14) Å	µ = 0.63 mm^−^^1^
*c* = 8.3878 (5) Å	*T* = 293 K
*V* = 1204.88 (12) Å^3^	Prism, yellow
*Z* = 4	0.10 × 0.10 × 0.10 mm

### Data collection

**Table:**

Nonius KappaCCD diffractometer	447 reflections with *I* > 2σ(*I*)
Radiation source: fine-focus sealed tube	*R*_int_ = 0.024
graphite	θ_max_ = 25.3°, θ_min_ = 3.1°
φ scans	*h* = −7→7
1080 measured reflections	*k* = −25→25
636 independent reflections	*l* = −10→10

### Refinement

**Table:**

Refinement on *F*^2^	Primary atom site location: structure-invariant direct methods
Least-squares matrix: full	Secondary atom site location: difference Fourier map
*R*[*F*^2^ > 2σ(*F*^2^)] = 0.055	Hydrogen site location: inferred from neighbouring sites
*wR*(*F*^2^) = 0.175	H-atom parameters constrained
*S* = 1.05	*w* = 1/[σ^2^(*F*_o_^2^) + (0.1002*P*)^2^ + 1.9948*P*] where *P* = (*F*_o_^2^ + 2*F*_c_^2^)/3
636 reflections	(Δ/σ)_max_ = 0.004
49 parameters	Δρ_max_ = 0.44 e Å^−^^3^
0 restraints	Δρ_min_ = −0.51 e Å^−^^3^

### Special details

**Table:**

Geometry. All e.s.d.'s (except the e.s.d. in the dihedral angle between two l.s. planes) are estimated using the full covariance matrix. The cell e.s.d.'s are taken into account individually in the estimation of e.s.d.'s in distances, angles and torsion angles; correlations between e.s.d.'s in cell parameters are only used when they are defined by crystal symmetry. An approximate (isotropic) treatment of cell e.s.d.'s is used for estimating e.s.d.'s involving l.s. planes.
Refinement. Refinement of *F*^2^ against ALL reflections. The weighted *R*-factor *wR* and goodness of fit *S* are based on *F*^2^, conventional *R*-factors *R* are based on *F*, with *F* set to zero for negative *F*^2^. The threshold expression of *F*^2^ > σ(*F*^2^) is used only for calculating *R*-factors(gt) *etc*. and is not relevant to the choice of reflections for refinement. *R*-factors based on *F*^2^ are statistically about twice as large as those based on *F*, and *R*- factors based on ALL data will be even larger.

### Fractional atomic coordinates and isotropic or equivalent isotropic displacement parameters (Å^2^)

**Table:**

	*x*	*y*	*z*	*U*_iso_*/*U*_eq_	Occ. (<1)
Cl1	0.0000	0.22960 (8)	0.7500	0.0492 (6)	
S1	0.5000	0.32187 (9)	0.7500	0.0582 (7)	
S2	0.0000	−0.02324 (10)	0.7500	0.1135 (15)	
N1	0.5000	0.21069 (18)	0.6134 (5)	0.0424 (10)	
H1	0.5000	0.2302	0.5240	0.051*	
N2	0.0000	0.0864 (2)	0.6144 (5)	0.0558 (12)	
H2A	0.0000	0.1262	0.6154	0.067*	
H2B	0.0000	0.0669	0.5251	0.067*	
C1	0.5000	0.1485 (2)	0.6079 (6)	0.0423 (11)	
C2	0.0000	0.0553 (4)	0.7500	0.0526 (19)	
C3	0.5000	0.1172 (3)	0.7500	0.0448 (17)	
H3	0.5000	0.0741	0.7500	0.054*	
C4	0.5000	0.2456 (3)	0.7500	0.0431 (16)	
C5	0.5000	0.1187 (3)	0.4493 (6)	0.0589 (15)	
H5A	0.5000	0.1499	0.3679	0.088*	
H5B	0.3821	0.0934	0.4385	0.088*	0.50
H5C	0.6179	0.0934	0.4385	0.088*	0.50

### Atomic displacement parameters (Å^2^)

**Table:**

	*U*^11^	*U*^22^	*U*^33^	*U*^12^	*U*^13^	*U*^23^
Cl1	0.0583 (11)	0.0481 (11)	0.0412 (10)	0.000	0.000	0.000
S1	0.0680 (13)	0.0463 (11)	0.0602 (14)	0.000	0.000	0.000
S2	0.267 (5)	0.0420 (14)	0.0316 (11)	0.000	0.000	0.000
N1	0.048 (2)	0.052 (3)	0.0276 (19)	0.000	0.000	0.0029 (18)
N2	0.084 (3)	0.053 (3)	0.030 (2)	0.000	0.000	0.0004 (19)
C1	0.048 (3)	0.046 (3)	0.033 (3)	0.000	0.000	0.000 (2)
C2	0.078 (5)	0.053 (4)	0.027 (4)	0.000	0.000	0.000
C3	0.059 (4)	0.041 (4)	0.035 (4)	0.000	0.000	0.000
C4	0.036 (3)	0.053 (4)	0.040 (4)	0.000	0.000	0.000
C5	0.086 (4)	0.063 (3)	0.028 (3)	0.000	0.000	−0.007 (2)

### Geometric parameters (Å, °)

**Table:**

S1—C4	1.649 (7)	C1—C5	1.479 (7)
S2—C2	1.698 (8)	C2—N2^i^	1.322 (6)
N1—C1	1.345 (6)	C3—C1^i^	1.371 (6)
N1—C4	1.371 (5)	C3—H3	0.9300
N1—H1	0.8600	C4—N1^i^	1.371 (5)
N2—C2	1.322 (6)	C5—H5A	0.9600
N2—H2A	0.8600	C5—H5B	0.9600
N2—H2B	0.8600	C5—H5C	0.9600
C1—C3	1.371 (6)		
			
C1—N1—C4	125.3 (4)	C1^i^—C3—C1	120.8 (6)
C1—N1—H1	117.4	C1^i^—C3—H3	119.6
C4—N1—H1	117.4	C1—C3—H3	119.6
C2—N2—H2A	120.0	N1^i^—C4—N1	113.3 (6)
C2—N2—H2B	120.0	N1^i^—C4—S1	123.3 (3)
H2A—N2—H2B	120.0	N1—C4—S1	123.3 (3)
N1—C1—C3	117.7 (5)	C1—C5—H5A	109.5
N1—C1—C5	117.8 (4)	C1—C5—H5B	109.5
C3—C1—C5	124.5 (5)	H5A—C5—H5B	109.5
N2^i^—C2—N2	118.8 (7)	C1—C5—H5C	109.5
N2^i^—C2—S2	120.6 (3)	H5A—C5—H5C	109.5
N2—C2—S2	120.6 (3)	H5B—C5—H5C	109.5
			
C4—N1—C1—C3	0.000 (1)	C5—C1—C3—C1^i^	180.0
C4—N1—C1—C5	180.000 (1)	C1—N1—C4—N1^i^	0.000 (2)
N1—C1—C3—C1^i^	0.000 (2)	C1—N1—C4—S1	180.0

Symmetry codes: (i) *x*, *y*, −*z*+3/2.

### Hydrogen-bond geometry (Å, °)

**Table:**

*D*—H···*A*	*D*—H	H···*A*	*D*···*A*	*D*—H···*A*
N1—H1···Cl1^ii^	0.86	2.46	3.310 (4)	171
N2—H2A···Cl1	0.86	2.50	3.297 (5)	154
N2—H2B···S2^iii^	0.86	2.49	3.347 (5)	173

Symmetry codes: (ii) −*x*+1/2, −*y*+1/2, −*z*+1; (iii) −*x*, −*y*, −*z*+1.
